# *Plasmodium vivax* congenital malaria in an area of very low endemicity in Guatemala: implications for clinical and epidemiological surveillance in a malaria elimination context

**DOI:** 10.1186/1475-2875-11-411

**Published:** 2012-12-08

**Authors:** María Eugenia Castellanos, Azucena Bardají, Michela Menegon, Alfredo Mayor, Meghna Desai, Carlo Severini, Clara Menéndez, Norma Padilla

**Affiliations:** 1 Centro de Estudios en Salud, Universidad del Valle de Guatemala (CES-UVG), 18 avenida 11-95 zona 15 Vista Hermosa 3, Guatemala, Guatemala; 2 Barcelona Centre for International Health Research, (CRESIB, Hospital Clínic- Universitat de Barcelona), Roselló, 132, 5-1, 08036, Barcelona, Spain; 3 Istituto Superiore di Sanità (ISS), Viale Regina Elena 299, 00161, Rome, Italy; 4 Centers for Disease Control and Prevention (CDC), Center for Global Health, Division of Parasitic Diseases and Malaria, 4770 Buford Hwy NE, Mail Stop F-22, Atlanta, GA, 30341, USA

**Keywords:** *Plasmodium vivax*, Congenital, Malaria, Guatemala

## Abstract

This is a report of the first *Plasmodium vivax* congenital malaria case in Guatemala and the first case in Latin America with genotypical, histological and clinical characterization. The findings show that maternal *P*. *vivax* infection still occurs in areas that are in the pathway towards malaria elimination, and can be associated with detrimental health effects for the neonate. It also highlights the need in very low transmission areas of not only maintaining, but increasing awareness of the problem and developing surveillance strategies, based on population risk, to detect the infection especially in this vulnerable group of the population.

## Background

Congenital malaria (CM) has been an often neglected consequence of malaria in pregnancy. It occurs when malaria parasites cross the placenta either during pregnancy or at delivery [1-3]. There is not much consensus regarding its definition. It has been defined as the presence of asexual parasites in the neonate’s peripheral blood within the first seven days of life or later if there is no exposure to infected mosquito bites, with or without clinical symptoms or signs [4]. Also, CM is often reported as the presence of malaria parasites in cord blood [3].

Congenital malaria has been predominantly reported for *Plasmodium falciparum* and from sub-Saharan Africa [5-7]. The precise prevalence of CM remains unclear, as it varies widely across endemic areas, ranging from 0.2% to 46.7% [5,6,8]. The use of PCR methods have revealed a higher frequency than it was previously reported [9-12]. Little information exists on its consequences for infant’s health, although it has been described that CM may lead to increased morbidity and mortality of the newborn if not accurately diagnosed and treated [13,14]. Moreover, prompt and effective malaria treatment during pregnancy has proven to be a protective factor for CM [14].

In Latin America, reports on CM have been scarce [9,15,16]; also, the effects of *Plasmodium vivax* congenital mono-infection on the neonate’s health have not yet been reported. This is the first *P*. *vivax* CM case from Guatemala and the first case in Latin American in which a comprehensive clinical, histological and genotypical investigation has been carried out.

## Case report

On September 2010, a 21-days-old female baby with a two-day history of fever was admitted to Fray Bartolomé de las Casas (FBC) District Hospital. FBC is a rural low-transmission malaria endemic area (population 55,073 inhabitants) in Northern Guatemala. FBC was a high-risk area where malaria transmission has been greatly reduced over the last years due to a scale-up of control interventions. Long-lasting insecticide-treated nets (LLITNs) and the improvement of the case management system implemented from 2006, resulted in a dramatic reduction in malaria parasite incidence from 91/1,000 population in 2006 to 1.9/1,000 population in 2010. Since 2009, no case of *P*. *falciparum* has been reported in the FBC area (National Malaria Control Program, Guatemala; unpublished data). Malaria transmission is sustained in the population by the temporal succession of the main vectors, *Anopheles albimanus*, *Anopheles vestitipennis* and *Anopheles darlingi*[17].

On admission physical examination of the baby revealed paleness, hepatosplenomegaly (1 cm), dusky skin appearance and desquamation, and a body temperature of 36.7°C which increased to 38.9°C in the next 24 hours. Neurological, respiratory and cardiac examinations were unremarkable. Main laboratory findings were anaemia (Hb 7.2 g/dL), severe thrombocytopaenia (45,000 platelets/μL on the 1^st^ day of admission, 24,000 platelets/μL on the 2^nd^ day of admission), and leucopaenia (white blood cells 3.5x10^3^/μL) with relative lymphocytosis (66.8%). Yet, the newborn did not show any signs or symptoms of haemodynamic failure nor evidence of bleeding throughout admission or after discharge. Biochemical and microbiological examinations were not available in the hospital. Based on initial suspicion of neonatal sepsis, the baby was started on intravenous (IV) ampicillin along with IV fluids and antipyretics. The thick blood smear examination showed *P*. *vivax* infection (parasite density 2,079 asexual parasites/μL), and then she was started on anti-malarial treatment with oral chloroquine (CQ) (¼ tablet of CQ 150 mg base, daily for five days). The neonate was treated with CQ only, following national guidelines in Guatemala that do not recommend the administration of primaquine to infants less than six months. Clinical progress was adequate and the baby was discharged after treatment completion. National guidelines also recommend therapy with ¼ tablet of CQ 150 mg base every 21 days until the patient is six months of age, so the infant kept on with CQ prophylaxis until that age after treatment completion. Follow-up during household visits two and eight months after hospital discharge was also favourable.

The 23-year-old primigravid mother was a FBC permanent resident. She was enrolled at the first antenatal clinic (ANC) visit, at 16 weeks of gestational age, in a multi-centre descriptive study –PregVax study- aimed to determine the burden and impact of *P*. *vivax* infection in pregnancy. As part of the PregVax study, maternal peripheral blood was collected at each ANC visit and at delivery for active detection of *Plasmodium* infection by microscopy and molecular methods. Samples were also collected from cord, placental, and newborn’s peripheral blood by heel prick at delivery for parasitaemia determination by microscopy and molecular methods, and placental impression smear and biopsy were taken for microscopic and histological examination, respectively.

This is a retrospective congenital malaria case report that mainly builds on the hospital clinical data available during its management. Molecular analyses, which were available due to the mother’s participation in the main study and carried out after the detection of CM infection, allowed the confirmation of the malaria case.

*Plasmodium* infection was not detected in the mother, either actively or passively, during pregnancy, neither by microscopy nor by polymerase chain reaction (PCR) at any of the ANC at 16, 21 and 26 weeks of gestational age. Delivery was at term (40 weeks), and the birth weight was 2,839 g. At delivery, the mother reported fever within the last 24 hours. Physical examination showed an axillary temperature of 38°C, paleness, and laboratory tests showed thrombocytopaenia (46,000 platelets/μL) and haemoglobin of 12.4 g/dL. *Plasmodium vivax* asexual parasites (6,429 parasites/μL) were detected in peripheral blood (see Table 1). She was treated with oral CQ (1,500 mg given over three days) and oral primaquine (15 mg daily for 14 days), following national guidelines. A follow-up home visit was conducted by the National Malaria Control Programme staff to assess treatment compliance and confirm parasite clearance.

**Table 1:** *Plasmodium vivax *parasitaemia and molecular results from the different compartments and time points

	**At birth**					**21 days after birth**
	**Mother at delivery**	**Cord**	**Placenta**		**Newborn**	**Newborn**
			**Impression smear**	**Histology**		
**Microscopy** (parasites/μL blood)^1^	6429	50	Not assessable	No parasites nor malaria pigment	Negative	2079
**RT**-**PCR**^**2**,**3**^	+	+	+	Not available	Negative	+

^1^ Parasitaemia density was estimated using the patients white blood count.

^2^ RT = Real-time PCR: + presence of *P*. *vivax* DNA.

^3^ Molecular analyses were carried out after the detection of the congenital malaria case in hospital. All samples at delivery (maternal, cord, placental and newborn blood) and during admission (newborn) were tested for *P*.*falciparum* (microscopy and real-time PCR) and were negative.

A placental biopsy sample was collected at delivery from the maternal side of the placenta, kept at 4°C in neutral buffer formalin, processed for histological examination, and stained with haematoxylin and eosin as previously described [18]. No evidence of parasites or malaria pigment deposition was found in the histological examination. Placental impression smears were not assessable due to lack of quality of the samples.

Immediately after birth, physical examination of the newborn revealed no clinical abnormalities. No *Plasmodium* infection was detected in the blood thick smear collected at delivery from the newborn. Examination of the cord blood thick smear showed *P*. *vivax* asexual parasites (50 parasites/μL). Cord blood parasitaemia determination is not routinely done at this health facility. In this case, cord blood parasitaemia determination was done later after birth at the study referral laboratory as part of the study objectives.

**Figure 1:**
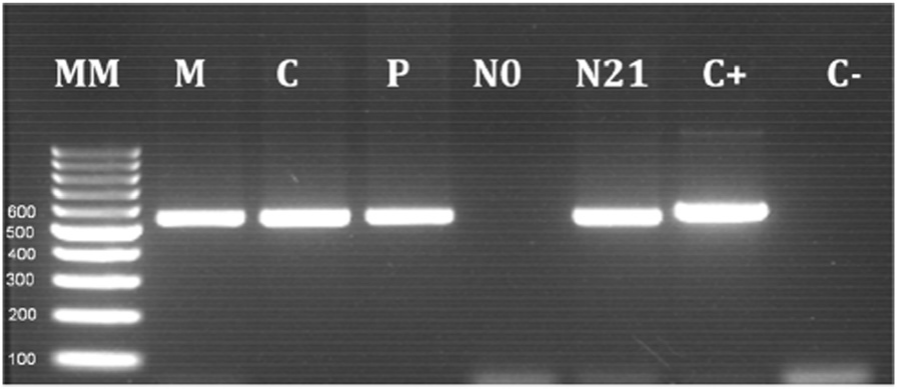
**Molecular detection of *Plasmodium vivax *in blood samples from mother and newborn by the amplification of *Pvmsp1 *gene.** PCR products were separated by electrophoresis through a 2.2% agarose gel stained with ethidium bromide and flanked by a 100 bp DNA ladder (Fermentas) as size marker. **Lane MM** = Molecular marker 100 bp. **Lane M** = Maternal peripheral blood at delivery. **Lane C** = Umbilical cord blood. **Lane P** = Placental blood. **Lane N0** = Newborn's blood at birth. **Lane N21** = Newborn’s blood at 21 days of life. **Lane C** + = Positive Control. **Lane C**- = Negative Control.

DNA extraction (Purelink TM Genomic DNA Kit – Invitrogen) was done on one whole blood-spot on filter paper from each of the following compartments: maternal peripheral blood at delivery, placental and cord blood, and neonate’s blood at the time of birth and during hospital admission. *Plasmodium vivax* infection was detected by conventional PCR for specific *P*. *vivax* merozoite surface protein 1 gene (*Pvmsp1*) in all samples except in the newborn’s blood at the time of birth (Figure 1) [19]. Also, confirmation of the presence of *P*. *vivax* and absence of *P*. *falciparum* infection was carried out on all samples by Real-Time PCR with a LightCycler® 480 system (Roche). Species-specific primers and probes were used as previously described by Veron *et al*. [20], selected from sequence of the small subunit of 18S rRNA, according to the following steps: pre-incubation at 95°C for 10 min; amplification at 95 °C for 10 sec, 50°C for 20 sec and 72°C for 5 sec for 50 cycles. All reactions were done in duplicate in a final volume of 20 μL.

Seven *P*. *vivax* microsatellites (MS) were amplified from *P*. *vivax* positive samples as described in Karunaweera *et al*. [21]. Briefly, PCR amplifications were performed in a 10-μL reaction mixture containing 2 μL of DNA, 1x reaction buffer, 1.75 mM MgC1_2_, 200 μM of each deoxynucleotide triphosphate, 5 picomoles of each primer and 2.5U of FastStar Taq polymerase (Roche). To determine the amplicon length variation, PCR products were analyzed by CEQ 8000 Genetic Analysis System (Beckman Coulter) using CEQ 8000 software for fragment analysis. Identical allelic profiles were obtained at the seven MS loci tested (MS1:231 bp, MS2:210 bp, MS3:177 bp, MS7:139 bp, MS8:243 bp, MS10:222 bp, MS20:187 bp), confirming that the same genotype was present in all compartments.

## Discussion

This is the first reported case of *P*. *vivax* congenital malaria in Guatemala and the first one from Latin America with clinical, histological and genotypical characterization. Co-infection with *P*. *falciparum* was excluded by molecular diagnosis, and the same parasite genotype in maternal blood at delivery, in placental and cord blood and in peripheral blood of the 21-days-old neonate was detected by microsatellite genotyping.

Several reports have been published from Latin America on malaria infection during the neonatal period [15,22,23] most of them due to *P*. *vivax*, but only three were reported as CM cases [9,15,16]. In none of these cases co-infection with other *Plasmodium* species was excluded. Most of the information on CM comes from high malaria transmission areas and indicates that CM is rarely symptomatic [3]. Although CM may be associated with non-specific symptoms shortly after birth, in most cases the infection is cleared spontaneously without symptoms. On rare occasions, clinical manifestations may appear within 10 to 42 days after birth [3]. In high endemic areas, passive transfer of maternal anti-malarial antibodies and the presence of foetal haemoglobin are thought to prevent disease by helping clearing parasitaemia in the newborn [4].

In the case outlined here, the onset of symptoms occurred 21 days after birth. The possibility that the neonate’s infection was acquired in the short period after birth from an infected mosquito bite is very low given the low malaria transmission in the FBC area. The microsatellite genotyping showing identical allelic profiles at all MS loci tested does reinforce the vertical transmission of the *P*. *vivax* infection, however, the possibility of a new infection, although very unlikely, it cannot be totally ruled out.

The physiopathology of CM is not well known. It has been hypothesized that, although the placenta functions as an effective barrier, maternal micro-transfusions into the foetal circulation, and direct penetration of the parasite through the placental villi could lead to vertical transmission of malaria [3]. In this case, vertical transmission is likely to have occurred around delivery, since the mother had symptoms at that time and parasitaemia was detected in maternal and cord blood [24]. The histological evaluation of the placenta did not reveal the presence of parasites or pigment, nor inflammation. The lack of placental features associated to malaria infection could be explained by the low density of maternal *P*. *vivax* infection and/or by the absence of a placental binding phenotype of *P*. *vivax* parasites [25].

In a malaria pre-elimination context, as is the case of the FBC area, the level of acquired anti-malarial immunity in the population may decreased and waned dramatically leading to increased susceptibility and risk of more severe forms of malaria when infection occurs. This may be particularly frequent and serious in vulnerable groups of the population, such as pregnant women and neonates. In the current case, the baby presented with severe thrombocytopaenia and anaemia, both being potential life-threatening haematological complications. This report highlights the need of establishing epidemiological and clinical surveillance strategies as malaria control programmes are reducing transmission to near-elimination levels, to effectively and promptly detect infections [26,27]. The latter would have avoided that malaria infection in this neonate would have developed into a life-threatening condition. These measures could include, firstly increasing clinical awareness since congenital malaria may not be considered a cause of illness in the neonate in such areas, and secondly, the identification of the most adequate strategy for active surveillance. This strategy would need to be evidence-informed on the population risk and based on a cost-effectiveness approach. Research needs to be done assessing the feasibility and cost-effectiveness of strategies, such as screening for all febrile babies and follow-up of those born to mothers who had malaria during pregnancy, whom should not be so many in low endemic areas.

## Conclusion

Mother-to-child transmission of *P*. *vivax* malaria occurs in very low malaria endemic settings, and it may be detrimental for the infant’s health. These findings emphasize the relevance in very low transmission areas of not only maintaining, but even increasing clinical and epidemiological awareness of this preventable and treatable disease in pregnancy and in the neonate. They also highlight the need for establishing active surveillance strategies in the areas that are in the pathway towards malaria elimination.

## Consent

The case study protocol was approved by the Universidad del Valle de Guatemala Ethics Review Committee. Written informed consent was obtained from the mother for her participation and that of her baby for the publication of this case report. A copy of the written consent is available for review by the Editor-in-Chief of this journal.

## Competing interests

The authors declare that they have no competing interests.

## Authors’ contributions

All authors have made substantial contributions to the investigations presented in this manuscript. MEC supervised all data collection and participated in drafting the manuscript, AB drafted the manuscript, and NP conceived and designed the report. MM and CS carried out the molecular and genotyping studies. AB, NP, AM, MD and CM revised the manuscript. All authors have read and approved the final manuscript.
